# National Association Railway Surgeons

**Published:** 1891-03

**Authors:** A. G. Gumear

**Affiliations:** Cor. Secretary, Buffalo, N. Y.


**Meeting of the National Association of Railway Surgeons.**—At the Kansas City meeting of the National Association of Railway Surgeons last year, it was decided to hold the next meeting at Buffalo May 7th, 8th and 9th of this year. But, on account of the meeting of the American Medical Association being set for the same time, it has been decided to change those dates, and to hold our next meeting at Buffalo April 30th and May 1st and 2d, to which all Railway Surgeons are cordially invited. To all Railway Surgeons sending their names and addresses to the Corresponding Secretary, a copy of the constitution and program will be sent. All those wishing to read papers should send in the titles of their papers without delay. For further information enquire of A. G Gumear, M. D.,
Cor. Secretary, Buffalo, N. Y.

